# Update on the Geographic Distribution of the Intermediate Host Snails of *Schistosoma mansoni* on St. Lucia: A Step Toward Confirming the Interruption of Transmission of Human Schistosomiasis

**DOI:** 10.4269/ajtmh.23-0235

**Published:** 2023-08-14

**Authors:** Samson Mukaratirwa, Martina R. Laidemitt, Reynold Hewitt, Mita E. Sengupta, Silvia Marchi, Consortia Polius, Sharon Belmar, Ronaldo G. C. Scholte, Freddy Perez, Anna-Sofie Stensgaard, Birgitte J. Vennervald, Arve L. Willingham, Eric S. Loker

**Affiliations:** ^1^ One Health Center for Zoonoses and Tropical Veterinary Medicine, Ross University School of Veterinary Medicine, Basseterre, Saint Kitts and Nevis;; ^2^ Parasitology Division, Museum of Southwestern Biology, Department of Biology, Center for Evolutionary and Theoretical Immunology, University of New Mexico, Albuquerque, New Mexico;; ^3^ Department of Veterinary and Animal Sciences, Faculty of Health and Medical Sciences, University of Copenhagen, Denmark;; ^4^ Ministry of Agriculture, Fisheries, Forestry, Food Security and Rural Development, Union, Castries, Saint Lucia;; ^5^ Ministry of Health, Wellness and Environment, Castries, Saint Lucia;; ^6^ Pan American Health Organization/World Health Organization, Washington, District of Columbia;; ^7^ Department of Veterinary Medicine, College of Agriculture and Veterinary Medicine, United Arab Emirates University, Al Ain, United Arab Emirates

## Abstract

To provide information to guide considerations of declaring interruption of transmission of human schistosomiasis due to *Schistosoma mansoni* on St. Lucia, we undertook an island-wide survey in June–July 2022 to determine the presence of *Biomphalaria* snails, the intermediate hosts of *S. mansoni*, and their infection status. Snail surveys were carried out at 58 habitats to determine presence of *Biomphalaria* snails followed by examination of the collected snails for evidence of infection with *S. mansoni*. Furthermore, water samples were collected at the snail habitats and screened for presence of *S. mansoni* DNA using an eDNA approach. We found *B. glabrata* present in one habitat (Cul de Sac) where it was abundant. Specimens provisionally identified as *Biomphalaria kuhniana* were recovered from 10 habitats. None of the *Biomphalaria* specimens recovered were positive for *S. mansoni*. None of the eDNA water samples screened were positive for *S. mansoni*. Experimental exposures of both field-derived and laboratory-reared St. Lucian *B. glabrata* and *B. kuhniana* to Puerto Rican and Kenyan-derived *S. mansoni* strains revealed *B. glabrata* to be susceptible to both and *B. kuhniana* proved refractory from histological and snail shedding results. We conclude, given the current rarity of *B. glabrata* on the island and lack of evidence for the presence of *S. mansoni*, that transmission is unlikely to be ongoing. Coupled with negative results from recent human serological surveys, and implementation of improved sanitation and provision of safe water supplies, St. Lucia should be considered a candidate for declaration of interruption of human schistosomiasis transmission.

## INTRODUCTION

Beginning in the 1970s, tremendous strides were made by the St. Lucian government in collaboration with the Rockefeller Foundation in the control of human intestinal schistosomiasis caused by *Schistosoma mansoni*, and by 1981, the prevalence had been reduced from 17% to < 2%.^1^ This resulted in control activities being sharply reduced without elimination of the disease.^2^ Currently, the transmission of *S. mansoni* is still considered ongoing in St. Lucia,^3^^–^^5^ although a recent study by Gaspard et al.^6^ suggests that transmission may have been interrupted after finding no positive cases in schoolchildren living adjacent to potential transmission sites. The intermediate host snail, *Biomphalaria glabrata*, is assumed to be present because efforts to eliminate the snail using *Melanoides tuberculata*, an exotic snail species as a biological competitor agent, may not have been completely successful.^7^ Another potential intermediate host snail, *Biomphalaria straminea*, was reported for the first time on the island in 1992, although its role in the transmission of schistosomiasis in St. Lucia is unknown.^7^ Other *Biomphalaria* species such as *B. kuhniana*, known from other Caribbean islands, might also be present.^8^ Gaspard et al.^6^ have rightly pointed out that “the situation in St. Lucia presents an opportunity to develop and evaluate possible approaches for verifying interruption of transmission.” A first step is to assess the current state of *S. mansoni* infection on Saint Lucia, and this should include both in humans and the snail intermediate host, *B. glabrata* (and possibly other *Biomphalaria* species present).

Thus, the government of St. Lucia established a steering committee with representatives from national key stakeholders under the coordination of the Ministry of Health and Wellness with the assistance of the Pan American Health Organization to establish guidelines to confirm interruption of transmission based on available information.^6^ Hence, updated information on the occurrence and geographic distribution of the intermediate hosts of *S. mansoni* (*Biomphalaria* spp.) and their infection status, based on a national malacological survey, is necessary to inform the guidelines. This information combined with national schistosomiasis surveys focusing on schoolchildren and other risk groups will give a more complete picture of the island’s current schistosomiasis status, needed to form the basis for an informed decision going forward. The inclusion of surveillance of known or suspected intermediate hosts, and their infection status in schistosomiasis control programs has become more pertinent, especially in areas where near elimination status of schistosomiasis needs to be confirmed.^9^

To inform the St. Lucian government further with respect to the status of schistosomiasis on the island, we conducted a targeted national malacological survey in St. Lucia in June–July 2022 to determine the geographic distribution and infection status of intermediate host snail(s) of *S. mansoni*, and to ascertain whether transmission might be ongoing. Here, we present the results of our study including updated geographic distribution data and trematode infection status for relevant snail species, employing both classical and molecular-based identification criteria. The malacological survey was undertaken in parallel with an environmental DNA (eDNA)-based sampling program designed to detect *S. mansoni* eDNA in snail habitats, a method shown to be highly sensitive for the detection of *S. mansoni* even in low-transmission settings.^10^ Additionally, we also undertook experimental infections of *Biomphalaria* species recovered from St. Lucia with *S. mansoni* to assess their status as potential mediators of transmission. Results from this study will help form the basis for recommendations for future efforts to monitor the interruption of schistosomiasis transmission, potentially including declarations of elimination of *S. mansoni* in St. Lucia.

## MATERIALS AND METHODS

### Collection of snails and screening for trematode cercariae.

**Figure 1.**
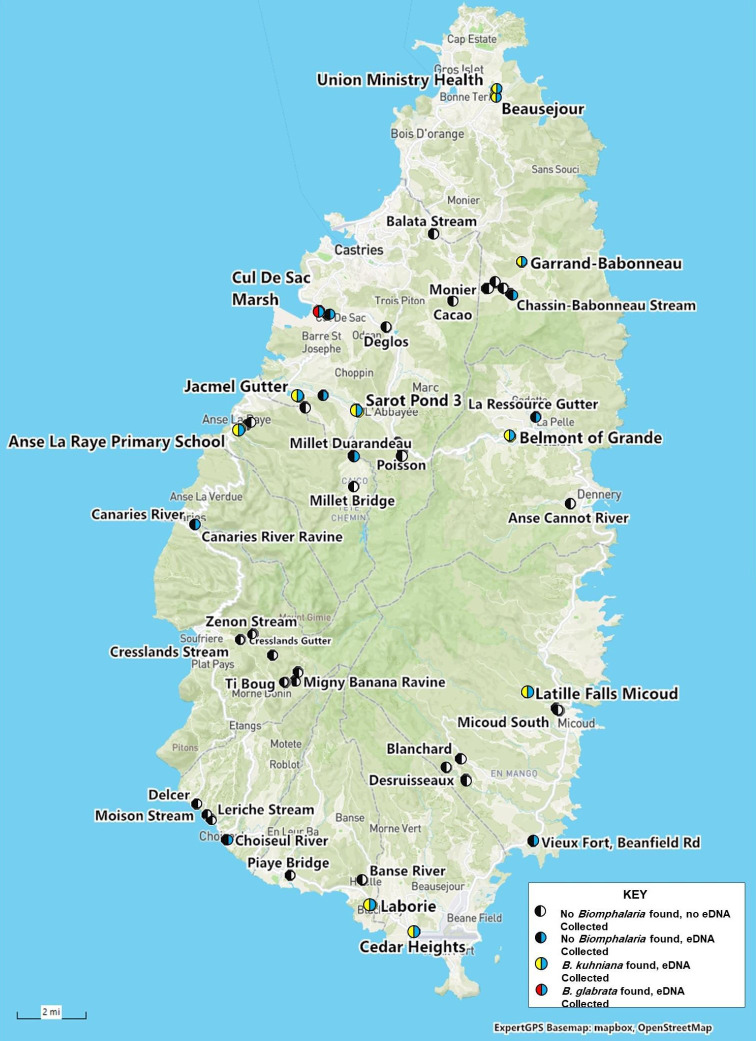
ExpertGPS basemap of St. Lucia and the habitats we sampled for aquatic snails. On the left side of the pie charts yellow indicates habitats that harbored *B. kuhniana*, red *B. glabrata*, and black no *Biomphalaria*. On the right side of the pie charts blue indicates eDNA samples were taken and white no samples were taken. See key on map for color coded pie charts.

We collected snails from 58 freshwater habitats on the island of St. Lucia between 25 June and 8 July 2022 (Figure 1 and Supplemental Table 1). Habitats that are overlapping in Figure 1 can be identified more fully in Supplemental File 1. At each habitat we recorded GPS coordinates, habitat type, distinctive features, the presence of people and/or animals, collected water samples appropriate for eDNA analysis (discussed subsequently), and then collected freshwater snails. Aquatic snails were collected along the water’s edge using kitchen sieves to sweep aquatic vegetation or a long-handled metal net to scoop along the substrate, rocks, and aquatic vegetation in deeper water. Snails were also picked off submerged rocks, plants, sticks, or debris using forceps. Collection at each habitat lasted between 30 minutes to 1 hour. Additional time was spent in the single habitat positive for *Biomphalaria glabrata*.

After collection, snails were cleaned with a Kimwipe™ (Kimberly-Clark Global Sales, LLC, Roswell, GA) to remove debris on their shells and then rinsed with clean water. Snails were placed individually into 12-well tissue culture plates in 3 mL of rainwater. Tissue culture plates were placed in ambient light for 1 hour to induce shedding of cercariae and were then screened for cercariae using an Olympus SZ61 (Tokyo, Japan) dissecting microscope. Keys based on shell or anatomic characters were used for the identification of snails^11^ and the cercariae they released.^12^^,^^13^ Snails and cercariae were preserved in 95% ethanol for later molecular analysis. Some snails were relaxed using menthol crystals, following the procedures of Pan,^14^ and were removed from their shells and fixed in Raillet–Henry’s solution to facilitate dissections and anatomical observations.

### Molecular characterization of *Biomphalaria* and trematodes.

One to three snails from habitats that harbored *Biomphalaria* were used for DNA extraction (SL1–SL15) (Table 1). A portion of the ovotestis cut from the whole snail using a razor blade was used for DNA extraction, and the rest of the snail was put in 95% ethanol and vouchered in the Museum of Southwestern Biology Parasite Division (MSB:HOST). Snail genomic DNA was extracted using the ENZA Mollusc Kit (Omega Bio-Tek, Norcross, GA). The elution volume was 60 μL, and the elution buffer was allowed to soak on the filter for 5 minutes before centrifugation. The 16S rRNA gene was chosen for amplification and sequencing due to the diversity of specimens in GenBank for Neotropical *Biomphalaria* for phylogenetic and genetic distance comparisons. Partial sequences of the 16S rRNA gene were amplified by polymerase chain reaction (PCR) using the primers published by Palumbi^15^: 16Sar, 5′-CGCCTGTTTATCAAAAACAT-3′ and 16Sbr, 5′-CCGGTCTGAACTCAGATCACGT-3′. The volume of each PCR was 25 μL, with 100 ng of DNA, 0.8 mM/L of deoxynucleotides, 2.5 mM/L of MgCl_2_, 0.25 units of TaKaRa Ex Taq DNA polymerase (Clontech, Mountain View, CA), and 0.15 μM/L of each primer. PCR cycles followed the study of Palumbi,^15^ with the exception of the annealing temperature of 44.1°C and run on an Eppendorf Mastercycler ep gradient S.

**Table 1:** *Biomphalaria* specimens and trematodes were chosen for DNA extraction and sequencing to determine species. Habitat, museum voucher numbers, and GenBank accession numbers are listed for each specimen

Sample	Species	Habitat	Musuem voucher	GenBank accession number(s)
SL1	*B. kuhniana*	Jacmel Gutter	MSB:Host:24883	OQ862773
SL2	*B. kuhniana*	Jacmel Gutter	MSB:Host:24878	OQ862774
SL3	*B. kuhniana*	Garrand-Babonneau	MSB:Host:24884	OQ862275
SL4	*B. kuhniana*	Anse La Raye Primary School Gutter	MSB:Host:24885	OQ862776
SL5	*B. kuhniana*	Anse La Raye Primary School Gutter	MSB:Host:24879	OQ862777
SL6	*B. kuhniana*	Union Ministry Health Ditch	MSB:Host:24887	OQ862778
SL7	*B. kuhniana* (juvenile)	Belmont of Grande Riviere	MSB:Host:24876	OQ862779
SL8	*B. kuhniana* (juvenile)	Belmont of Grande Riviere	MSB:Host:24880	OQ862780
SL9	*B. kuhniana*	Latille Falls Micoud	MSB:Host:24888	OQ862781
SL10	*B. kuhniana*	Beausejour	MSB:Host:24881	OQ862782
SL11	*B. kuhniana*	Beausejour	MSB:Host:24874	OQ862783
SL12	*B. glabrata*	Cul de Sac Marsh	MSB:Host:24875	OQ862784
SL13	*B. glabrata*	Cul de Sac Marsh	MSB:Host:24886	OQ862785
SL14	*B. kuhniana*	Laborie	MSB:Host:24882	OQ862786
SL15	*B. kuhniana* (Adult)	Belmont of Grande Riviere	MSB:Host:24877	OQ862787
SL16	Schistosomatidae E	Belmont of Grande Riviere	MSB:Para:35977	OQ868122; OQ866318
SL17	*Patagifer sp.* 2	Belmont of Grande Riviere	MSB:Para:35976	OQ868123; OQ868519
SL18	*Patagifer sp.* 2	Cul de Sac Marsh	MSB:Para:35978	OQ868124; OQ868518

Genomic DNA from one or two trematode cercariae collected from shedding snails (Table 1) were extracted using the Qiagen DNA Micro Kit (Qiagen, Valencia, CA) with a final elution volume of 35 μL. The 28S gene was amplified using forward primer, dig12 (5′-AAGCATATCACTAAGCGG-3′) and reverse primer 1500R (5′-GCTATCCTGAGGGAAACTTCG-3′).^16^ The volume of each PCR reaction was 25 μL with 2 μL of 50 ng of DNA, 0.8 mM/L dNTPs,2.5 mM/L MgCl_2_, 0.25 units of Ex Taq DNA (Clontech, Mountain View, CA), and 0.4 μM/L of each. The *cox*1 gene for the avian schistosome was amplified using the Schist 5′ and Schist 3′ primers.^17^ The *nad*1 gene for the echinostomes were amplified using the forward primer NDJ11 (5′-AGA TTCGTA AGG GGC CTA ATA-3′) and the reverse primer NDJ2a (5′-CTT CAG CCT CAG CAT AAT-3′)^18^ with the same PCR setup as the 28S gene. See Laidemitt et al.^19^ for PCR profiles of the *nad*1 and 28S genes.

PCR products were separated by agarose gel electrophoresis, visualized with a 0.5% GelRed nucleic acid gel stain (Biotium Inc., Hayward, CA) and were purified using ExoSap-IT (Applied Biosystems, Foster City, CA). Both strands were sequenced using an Applied Biosystems 3130 automated sequencer and BigDye Terminator Cycle Sequencing Kit version 3.1 (Applied Biosystems). DNA sequences were verified by aligning reads from the 5′ and 3′ directions using Sequencher 5.1 and manually corrected for ambiguous base calls (Gene Codes, Ann Arbor, MI). Approximately 460 bases were generated of the 16S rRNA gene. Sequences were aligned by CLUSTAL W, and the best fit model of substitution was modeled in Molecular Evolutionary Genetics Analysis 11 (MEGA11).^20^ Phylogenetic analyses using maximum likelihood (ML) included our 15 samples along with 56 sequences from the National Center for Biotechnology Information-GenBank for 16S. A total of 460 positions were used and along with Heuristic searches, 1,000 bootstrap replicates were run in MEGA11 for ML analysis. Uncorrected pairwise distance values (*P* values) were calculated in MEGA11, and a > 5% in mtDNA difference between samples was used to provisionally delineate species.^21^^,^^22^

### Detecting *S. mansoni* in snails using a nad5 PCR assay.

A nicotinamide adenine dinucleotide dehydrogenase subunit 5 (*nad*5) PCR assay^23^ was used on the extracted DNA *Biomphalaria* samples (SL1–SL15) to determine if there were prepatent *S. mansoni* infections that could be detected by PCR. This is a sensitive assay (> 0.1 fg DNA) and differentiates *Schistosoma* species either by band size or absence/presence. We followed the same PCR and gel imaging protocol as Lu et al.^23^ except we used TaKaRa Ex Taq DNA^®^ polymerase, buffer, and dNTPs (Clontech, Mountain View, CA). For a positive control, we used DNA from one *Biomphalaria glabrata* shedding *S. mansoni.*

### Sampling and analysis of water to detect *S. mansoni* eDNA.

Water was collected from the snail habitats by a person designated to collect water samples for eDNA analysis, before anyone disturbing the habitat. This was done to avoid any potential contamination with parasite DNA of the water sample. A representation of the collected samples (Figure 1) covering the habitats where *Biomphalaria* snails were collected as well as geographically spread out on St. Lucia were selected for further eDNA analysis. Three replicates of 500 mL of water were collected from each habitat, with several distinct areas (both sides of a stream, vegetated and unvegetated, etc.) per habitat depending on the total area of each habitat. Bottles containing water samples were labeled appropriately and kept cool until returned to the laboratory, where the water sample was filtered the same day as collection. To capture eDNA, each sample was filtered using a vacuum pump (Chemical, Duty Pump, Millipore, Billerica, MA) through a 0.45-µm disc-filter (Whatman^®^ glass microfiber filters WHA1825047, or Whatman (Sigma-Aldrich, Milwaukee, WI) membrane filters 7140-104). If the water was extremely turbid, a larger pore filter of 2.7 µm was used (Whatman glass microfiber filters, WHA1823047). The filter was then removed and placed in a separate LoBind 2-mL Eppendorf tube with RNAlater and held at room temperature until the filter was prepared for eDNA analysis. The entire filtering apparatus and collection bottles were cleaned with 5% bleach and rinsed three times between samples to avoid cross contamination.

In brief, DNA was extracted from the filters using the DNeasy Blood and Tissue Kit (Qiagen) following a modified protocol for water eDNA samples, as described in Spens et al.^24^ Extraction blanks were included for all extractions. A qPCR-assay with species-specific primers/probe targeting *S. mansoni* was used for analyzing the samples in PCR triplicates, following Sengupta et al.,^10^ where details on PCR mastermix, and thermal settings are found. Negative controls (NTC) were included for all qPCR runs. Filters with *S. mansoni* cercariae ranging from 1 to 25 cercariae were used as positive controls and DNA was extracted from the filters and analyzed with the *S. mansoni* specific quantitative PCR assay in the same way as the water samples. Lastly, to check for inhibition in the water samples, an internal positive control (TaqMan Exogeneous Internal Positive Control) was included for all samples.

### Experimental exposures of field-derived and laboratory-reared St. Lucian *Biomphalaria* to *S. mansoni.*

*Biomphalaria glabrata* from Cul de Sac and *B. kuhniana* from Anse la Raye Primary School, Beausejour, and Belmont Grande of Riviere collecting habitats were used to establish laboratory colonies at the University of New Mexico (UNM). Both field-caught and F1 laboratory-reared snails (all initially negative for schistosome or other trematode infections as ascertained by lack of shedding cercariae) were exposed to miracidia of the Puerto Rico 1 (PR1) strain of *S. mansoni* originally sourced from the Schistosomiasis Resource Center, Biomedical Research Institute, Rockville, MD. Additionally, F1 generation snails were exposed to miracidia from a Kenyan isolate of *S. mansoni* maintained since 2013 at UNM in *Biomphalaria choanomphala* and hamsters. All snails were individually exposed to 10 freshly pipetted (hatched within 30 min) miracidia derived from livers of experimentally infected mice or hamsters and were maintained at 24°C, 12 h light:12 h dark cycle, fed red leaf lettuce ad libitum and shrimp pellets twice a week. They were examined for evidence of release of *S. mansoni* cercariae by isolating each snail in a well of a 12-well cell culture plate with 3 mL of artificial spring water and placing them under indirect light for 2 hours at 4.5, 5.5, 6.5, and 7.5 weeks post-exposure (WPE). All exposed snails were dissected after 7.5 WPE. Vertebrate animal use for this study was approved by the UNM Institutional Animal Care and Use Committee (IACUC 22-201290-MC).

In addition, some snails exposed to *S. mansoni* for 1, 2, or 4 days were prepared for histological examination. Snails were placed in Railliet–Henry’s fixative for at least 48 hours. The shell of each snail was removed, and the head-foot of the snail was placed in 10% neutral buffered formalin. The snails were processed at TriCore Reference Laboratories in Albuquerque, New Mexico, sectioned, and sections stained with hematoxylin and eosin.

## RESULTS

### Snail and trematode molecular results.

**Figure 2.**
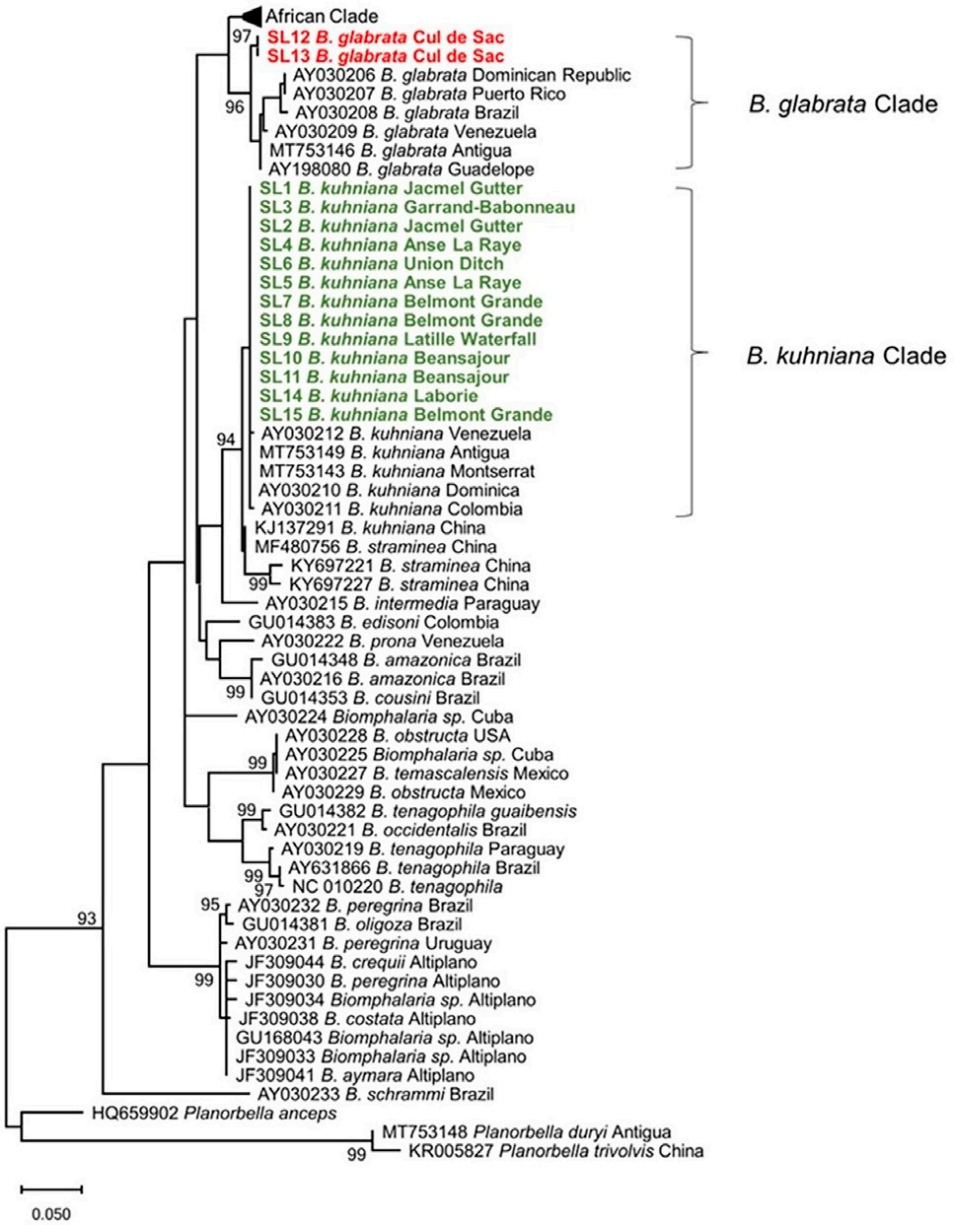
Evolutionary analysis by maximum likelihood (ML). The ML tree was based on 460 positions of the 16S rRNA gene from 15 specimens of *Biomphalaria* collected in this study (bolded) and 56 specimens from GenBank. The *B. glabrata* specimens are in red and the *B. kuhniana* specimens are in green. Sister species in the genera *Planorbella* were chosen for the outgroup. A total of 1,000 bootstraps were run, and model GTR + I + G was selected via model selection. The percentage of trees in which the associated taxa clustered together is shown next to the branches (> 90%). Initial tree(s) for the heuristic search were obtained automatically by applying Neighbor-Join and BioNJ algorithms to a matrix of pairwise distances estimated using the maximum composite likelihood approach, and then selecting the topology with superior log likelihood value. The tree is drawn to scale, with branch lengths measured in the number of substitutions per site. Codon positions included were 1st+2nd+3rd+Noncoding. Evolutionary analyses were conducted in MEGA11.

Among the 58 habitats sampled (Figure 1), we collected freshwater snails representing at least 11 species, all of which were isolated and examined for trematode cercariae. All snail species and cercariae are reported in Supplemental Table 1. We recorded 659 *Biomphalaria* specimens from 11 habitats. Identification of the *Biomphalaria* snails was confirmed by examination of conchological features, dissections, and by sequence data for the 16S rRNA marker gene. *Biomphalaria glabrata* was recovered from a single habitat (Cul de Sac) (Figure 2). All other *Biomphalaria* collected had shell anatomy and size, dissected genitalia including numbers of prostate diverticuli, and sequence data consistent with *B. kuhniana* as described by Pointier.^11^

None of the *Biomphalaria* snails collected from St. Lucia in this study were infected with *S. mansoni*. One *B. kuhniana* from Belmont of Grande Ravine in the Parish of Dennery was shedding schistosome cercariae, which proved to have eyespots and to be cercariae of an avian-infecting schistosome species. Examination of 28S and *cox*1 gene markers indicated these St. Lucian avian schistosome cercariae were very similar to cercariae from *B. straminea* from Brazil designated as Schistosomatidae E.^25^ These cercariae are likely congeners to an avian schistosome (AY829246) from *Biomphalaria sudanica* from Kenya.^26^ One *B. kuhniana* from Belmont of Grande Ravine and three *B. glabrata* from Cul de Sac were shedding *Patagifer* sp. 2 (based on 28S and *nad*1 gene markers). This species is known to infect two *Biomphalaria* species in Kenya,^19^ and a similar cercaria has been reported from *Biomphalaria orbignyi* from Argentina.^27^ The genus is known for infecting ibises (subfamily Threskiornithinae) as definitive hosts and snails as first and second intermediate hosts.^28^ No other trematode infections were found among the St. Lucian *Biomphalaria* snails we sampled.

One of the most striking aspects of the freshwater snail fauna of St. Lucia is the extent to which it is dominated by the exotic snail, *Melanodies tuberculata*, which was collected from 49 of 58 habitats we sampled. In most places, particularly so for streams, it was by far the most abundant gastropod collected. Historically, before the introduction of *M. tuberculata*, *B. glabrata* was commonly recovered from streams. In the one marshy habitat where we found *B. glabrata*, *M. tuberculata* was also present but was not numerically dominant.

### *nad5* PCR assay.

No schistosome bands were detected in any of the 30 (four *B*. *glabrata* and 26 *B*. *kuhniana*) snails examined using this assay. Positive controls were positive, and negative controls were negative.

### *Schistosoma mansoni* eDNA results.

A total of 63 filtered water samples from 21 locations (Table 1) were analyzed for *S. mansoni* eDNA, and no amplification of *S. mansoni* DNA was detected, including the Cul de Sac habitat where *B. glabrata* snails were found. All negative controls throughout the analysis process, such as extraction blanks and PCR negative controls, for example, were negative, and all the positive controls were also positive. For the filters spiked with *S. mansoni* cercariae, the qPCR assay picked up an *S. mansoni* DNA signal on each filter, even the filter containing only one *S. mansoni* cercariae. Amplification of the internal positive control indicated that no inhibition was detected in the water samples.

### Experimental exposures.

**Figure 3.**
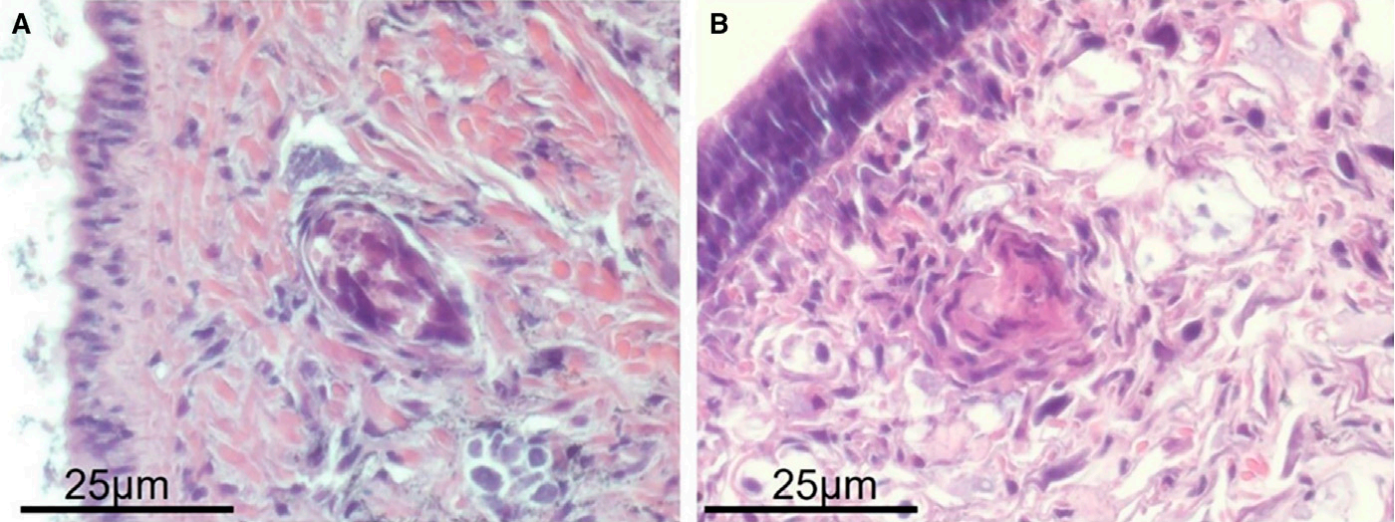
Histological sections of laboratory-reared *Biomphalaria kuhniana* from Anse La Raye, St. Lucia, exposed to 10 PR1 *S. mansoni* miracidia for 1 day post-exposure (DPE) (**A**) or 4 DPE (**B**). At 1 DPE, the sporocyst retains its anatomic integrity and a thin layer of hemocytes can be seen surrounding it. At 4 DPE, the sporocyst has been heavily encapsulated by host hemocytes and its anatomical integrity lost.

With respect to experimental exposures to *S. mansoni* (Table 2), *B. glabrata* from Cul de Sac proved to be susceptible to both PR1 (Puerto Rican) and Kenyan isolates of *S. mansoni*, particularly so to the isolate of Caribbean origin, which produced higher infection rates (61%) than the Kenyan *S. mansoni* isolate (31%) at 7.5 WPE. The *B. glabrata* exposed to the PR1 isolate also started shedding cercariae a week earlier (4.5 WPE) compared with the *B. glabrata* exposed to the Kenyan isolate (5.5 WPE). None of the exposed *B. glabrata* that failed to shed cercariae of *S. mansoni* were found positive upon dissection. Also, the number of cercariae shed per infected snail, although not enumerated, were conspicuously fewer in snails exposed to *S. mansoni* of Kenyan origin. None of the *B. kuhniana* (field-derived or laboratory-reared F1s including neonates) exposed to either isolate of *S. mansoni* shed any *S. mansoni* cercariae. Examination of histological sections showed that *S. mansoni* miracidia penetrated *B. kuhniana* (Figure 3A), but the only sporocysts seen by 4 days postexposure were encapsulated and had lost their typical anatomical structure (Figure 3B). All *B. kuhniana* exposed to *S. mansoni* were dissected 48 days postexposure and were negative for sporocysts of *S. mansoni*.

**Table 2:** Experimental exposure of *Biomphalaria* spp. with two strains of *Schistosoma mansoni*

Species	Habitat	Origin of *S. mansoni*	No. of miracidia	No. of snails exposed	4.5 WPE # infected/ survivors (% shedding)	5.5 WPE # infected/ survivors (% shedding)	6.5 WPE # infected/ survivors (% shedding)	7.5 WPE # infected/ survivors (% shedding)	Dissections
*B. glabrata* (field-dervied 10–12 mm)	Cul de Sac Marsh	Puerto Rico	10	22	2/20 (10%)	8/20 (40%)	9/20 (45%)	11/18 (61%) 2/18 shed *Patagifer*	Snails dissected—11 positive and shedding cercariae
*B. glabrata* (F1s 5–7 mm)	Cul de Sac Marsh	Kenya	10	22	0/18 (0%)	2/16 (12.5%)	4/16 (25%)	5/16 (31%)	Snails dissected—only five positive and shedding cercariae
*B. kuhniana* (field-derived 6-8 mm)	Belmont of Grande Riviere	Puerto Rico	10	24	0/23 (0%)	0/23 (0%)	0/23 (0%)	0/18 (0%)	Snails dissected negative for sporocysts or cercariae
*B. kuhniana* (neonates)	Belmont of Grande Riviere	Puerto Rico	5	24	0/22 (0%)	0/20 (0%)	0/14 (0%)	0/4 (0%)	Snails dissected negative for sporocysts or cercariae
*B. kuhniana* (field-derived 6–8 mm)	Anse La Raye	Puerto Rico	10	17	0/17 (0%)	0/17 (0%)	0/17 (0%)	0/15 (0%)	Snails dissected negative for sporocysts or cercariae
*B. kuhniana* (F1s 2–4 mm)	Anse La Raye	Kenya	10	58	0/33 (0%)	0/32 (0%)	0/31 (0%)	0/31 (0%)	Snails dissected negative for sporocysts or cercariae
*B. kuhniana* (F1s 2–4 mm)	Anse La Raye	Puerto Rico	10	48	0/36 (0%)	0/36 (0%)	0/36 (0%)	0/36 (0%)	Snails dissected negative for sporocysts or cercariae
*B. kuhniana* (Field-Derived 6–8 mm)	Stadium	Puerto Rico	10	24	0/22 (0%)	0/20 (0%)	0/20 (0%)	0/16 (0%)	Snails dissected negative for sporocysts or cercariae
*B. kuhniana* (F1s 2–4 mm)	Beausejour	Kenya	10	52	0/41 (0%)	0/36 (0%)	0/36 (0%)	0/35 (0%)	Snails dissected negative for sporocysts or cercariae
*B. kuhniana* (F1s 2–4 mm)	Beausejour	Puerto Rico	10	12	0/12 (0%)	0/12 (0%)	0/11 (0%)	0/11 (0%)	Snails dissected negative for sporocysts or cercariae

## DISCUSSION

*Biomphalaria glabrata* is now rare, but not absent, on St. Lucia. It was found in only one of the 58 habitats we sampled. Of the 26 habitats sampled by Pointier 1993,^7^ our study sampled 21 of them. Of the remaining five, although we could not pinpoint the original locations specified by Pointier, we sampled habitats that were spatially close to them (Supplemental Table 1). Of the 21 habitats, 14 formerly harbored *B. glabrata*. Compared with precontrol efforts when *B. glabrata* was widely distributed across the island, its present distribution as best we could determine is limited to a single, but fairly extensive, flat marshland in northcentral St. Lucia. This Cul de Sac habitat was problematic for schistosomiasis transmission in St. Lucia in the past and was the last habitat known to be treated by molluscicide, which occurred in 1988.^7^

None of the *Biomphalaria* snails we collected on St. Lucia were infected with *S. mansoni* by shedding or PCR. However, it is well known that the infection prevalence/shedding rate of schistosome intermediate hosts can be quite low (often less than 2%), compared with the infection rates sometimes observed in mammalian hosts.^29^ Therefore, absence of infected snails among our samples does not necessarily rule out transmission. For this reason, we also deployed eDNA, a method that since it was first developed for *S. mansoni*,^10^^,^^30^ has found use as a sensitive, noninvasive environmental monitoring tool in medical and veterinary parasitology.^31^ None of the eDNA samples collected and analyzed on St. Lucia were positive for *S. mansoni*, which further supports the absence of infected snails in the identified *Biomphalaria* snail habitats (Figure 1). Especially in a low-transmission setting, such as would be expected in St. Lucia, the fact that *S. mansoni* eDNA can persist for up to 8 days^10^ further supports that no recent shedding events have taken place in the examined snail habitats. The absence of eDNA signals, whether from miracidia or cercariae, also suggests the infection is not being maintained in reservoir hosts, although this is an area that might require further study.

Although we did not find any indication of ongoing *S. mansoni* transmission, our experimental infection results indicated *B. glabrata* of Cul de Sac origin is still compatible with *S. mansoni*, as tested with the PR1 isolate of *S. mansoni* derived from Puerto Rico, which has been maintained for decades in the laboratory. It is also susceptible, although less so, with an isolate of *S. mansoni* from Kenya. It might be from an immigrant of African or southwest Asian origin whereby *S. mansoni* could potentially be reintroduced into St. Lucia, so the susceptibility of local *B. glabrata* to a Kenyan isolate highlights a lingering possibility for such an event.

The snail taxon we identified as *B. kuhniana* was relatively widely distributed on St. Lucia. It is appropriately cautious to consider our identification of the St. Lucian *B. kuhniana* specimens as provisional because it is clear from both the literature,^32^^,^^33^ including a recent detailed study by de Araujo et al.,^34^ and from GenBank entries that *B. kuhniana*, *B. straminea*, and *B. intermedia* are closely related and may best be viewed as a complex of diverging species. Genetic distances between some isolates of provisional *B. kuhniana* and *B. straminea* fall in an ambiguous region of < 3% divergence. Specimens indistinguishable from *B. kuhniana* based on appearance and sequence of our chosen marker gene were also found on Antigua and Montserrat.^35^ Further collection of specimens from broader geographic areas and additional sequence data from more genes, including nuclear genes, will be required to resolve this matter fully.

We show here that provisional *B. kuhniana* is capable of naturally transmitting an avian schistosome on St. Lucia. We are not aware of any reports of *B. kuhniana* serving as a natural host for *S. mansoni*, and Paraense^32^^,^^36^ indicated this snail had proven refractory to *S. mansoni* infection. Pointier^11^ considered its status as a host for *S. mansoni* to be “unclear.” Our experimental infections using both PR1 and Kenyan isolates of *S. mansoni* indicated that miracidia were able to penetrate *B. kuhniana*, and evidence of encapsulation of sporocysts and dismemberment by hemocytes was observed. Upon dissection of exposed snails at 48 days postexposure, none of the *B. kuhniana* showed indication of *S. mansoni* sporocysts or cercariae within them. We conclude the status of provisional *B. kuhniana* as potential future hosts of *S. mansoni* in the Caribbean should not be overlooked, but based on results from this study, they appear to be incompatible with *S. mansoni* development. *Biomphalaria kuhniana* is also a potential competitor of *B. glabrata* responsible in part for the latter species decline in abundance in Martinique,^36^^,^^37^ and it is conceivable that in certain situations, if co-occurring with *B. glabrata*, it could act as a decoy host for miracidia of *S. mansoni*.

The current abundance of *Melanoides tuberculata* in almost all freshwater habitats on St. Lucia is remarkable, and it is certainly tempting to conclude that this species has played a major role in diminishing *B. glabrata* abundance on the island by playing a competitor role previously discussed by several authors.^7^^,^^11^^,^^38^^,^^39^ In a previous survey of 26 St. Lucian snail habitats, Pointier^7^ noted that 24 (92.3%) habitats contained *M. tuberculata*, and 19 (73.0%) harbored *B. glabrata*, with 17 (65.4%) containing both species. The two habitats where *B. glabrata* was most abundant lacked *M. tuberculata*. Comparison with our results suggests that *B. glabrata* has since become much less common on St. Lucia, and even the single habitat where we found *B. glabrata* also harbored *M. tuberculata*.

Although beneficial from the standpoint of schistosomiasis control in the short term, the presence of *M. tuberculata* is also reason for concern because it represents a major alteration to native St. Lucian freshwater biotas, similar to what has happened on Martinique.^36^ Such snails might begin to transmit trematodes of medical, veterinary, or conservation concern.^40^^,^^41^ Also, it should be noted that the flora and fauna of islands are subject to high immigration and extinction rates, so future changes in freshwater habitats in St. Lucia may be unpredictable, particularly considering changing climates.

In conclusion, our study provides no evidence for the continued presence of *S. mansoni* on St. Lucia. Both snail and eDNA surveys found no evidence for the presence of *S. mansoni*. Furthermore, compared with past surveys, we found that the primary snail vector of concern, *B. glabrata*, is now rare on St. Lucia, and that the other *Biomphalaria* species present, *B. kuhniana*, does not support the full intramolluscan development of *S. mansoni* culminating in production of human-infecting cercariae or patent infections. In fact, *B. kuhniana* seems to mount active resistance responses that destroy young *S. mansoni* sporocysts. We note that the susceptibility status for *S. mansoni* of the snails we provisionally identified as *B. kuhniana* could change, possibly influenced by its infection with other facilitating parasite species,^42^ or in response to relaxed selection for resistance owing to the present lack of *S. mansoni*. Additionally, while visiting freshwater habitats across the island, including Cul de Sac, we were struck by the limited extent to which St. Lucians now seem to depend on surface waters for bathing or clothes washing, thereby diminishing the likelihood of exposure to infection should the parasite be present. Finally, we note that the possibility remains that there are isolated foci of infection maintained by rodent or other reservoir hosts,^43^^–^^45^ though this seems less likely to be an issue of concern given the rarity of *B. glabrata*, the lack of supporting eDNA evidence, and the potential difficulty of short-lived rodents to maintain transmission under such circumstances.

## Supplemental Materials


Supplemental materials
