# Supplementary material for: Update on the Geographic Distribution of the Intermediate Host Snails of Schistosoma mansoni on St. Lucia: A Step Toward Confirming the Interruption of Transmission of Human Schistosomiasis
Source: Am J Trop Med Hyg. 2023 Aug 14;109(4):811–9. doi: 10.4269/ajtmh.23-0235 (PMC10551094; doi:10.4269/ajtmh.23-0235)
Supplement: Supplementary file 1 [file tpmd230235.SD1.pdf]

Supplementary Table 1. Malacological information from St. Lucia. In some cases, P is used to indicate a species was present in a habitat, but not necessarily enumerated.

**Habitat**	**Color on Map**	**Habitat Type**	**Date Collected**	**Latitude**	**Longitude**	***Biomphalaria* species (#) and Trematodes (#)**	***Physa***	***Melanoides***	***Drepanotrema***	***Neritina***	***Pseudosuccinea***	***Pomacea***	**Hydrobiidae**	**Water analyzed for *S. mansoni* eDNA**
Anse Cannot River	Black	River	30-Jun-22	13.90909	-60.8998	0	0	P	0	0	0	0	0	No (N)
Anse La Raye Care School	Black	Hydroponic apparatus	29-Jun-22	13.93864	-61.0384	0	15	0	0	0	0	0	0	N
Anse La Raye Primary School Gutter	Yellow	Drainage Gutter	29-Jun-22	13.93693	-61.0409	*kuhniana* (225)	3	P	0	0	0	0	0	Yes (Y)
Anse La Raye Primary School Pond*	Black	Pond	4-Jul-22	13.93693	-61.0409	0	0	P	0	0	0	0	0	N
Balata Stream	Black	Stream	27-Jun-22	14.01615	-60.958	0	0	0	0	0	0	0	0	N
Banse River	Black	River	4-Jul-22	13.75619	-60.989	0	0	P	0	0	0	0	0	N
Beausejour	Yellow	Pond	29-Jun-22	14.07188	-60.9315	*kuhniana* (91)	12	P	0	0	0	0	0	Y
Belmont of Grande Riviere*	Yellow	Water catchment	30-Jun-22	13.93381	-60.9248	*kuhniana* (260) avian schistosome (1), echinostome (1)	43	P	0	0	3	3	0	Y
Bexon River	Black	River	28-	13.93	-60.973	0	0	P	0	0	0	0	0	N

			Jun-22	131	3									
Blanchard*	Black	Dried River	4-Jul-22	13.80572	-60.9472	0	0	0	0	0	0	0	0	N
Babonneau River	Black	River	28-Jun-22	13.91614	-60.9931	0	0	P	0	0	0	0	0	N
Cacao*	Black	Stream	28-Jun-22	13.98902	-60.9496	0	P	P	0	0	0	0	0	N
Canaries River	Black	River	4-Jul-22	13.90075	-61.0616	0	0	P	0	P	0	0	0	N
Canaries River Ravine	Black	Ravine	4-Jul-22	13.90048	-61.0611	0	7	P	7	0	0	0	0	Y
Canaries River Ravine	Black	Gutter	4-Jul-22	13.90048	-61.0611	0	41	P	1	0	0	0	0	Y
Cedar Heights	Yellow	Pond	4-Jul-22	13.73556	-60.9669	*kuhniana* (1)	0	0	0	0	0	0	0	Y
Chassin-Babonneau	Black	River	28-Jun-22	13.99396	-60.9277	0	P	P	0	0	0	0	0	Y
Chassin-Babonneau	Black	Stream	28-Jun-22	13.99184	-60.925	0	0	P	0	0	0	0	0	N
Choiseul River	Black	River	4-Jul-22	13.77289	-61.0477	0	0	P	0	P	0	0	0	Y
Cresslands Gutter	Black	Gutter	30-Jun-22	13.8538	-61.0417	0	0	P	0	0	0	0	0	N

Cresslands Stream*	Black	Stream	30-Jun-22	13.8538	-61.0417	0	0	P	0	0	0	0	0	N
Cul de Sac Marsh*	Red	Marsh	5-Jul-22	13.9847	-61.007	*glabrata* (38), echinostome (3)	18	P	147	0	0	0	0	Y
Cul de Sac Stream*	Black	Stream	5-Jul-22	13.98519	-61.0073	0	P	0	4	P	0	0	2	Y
Cul de Sac West Side*	Black	Marsh	5-Jul-22	13.98317	-61.0037	0	9	0	45	0	0	0	0	Y
Deglos*	Black	Water catchment	29-Jun-22	13.97814	-60.9782	0	0	P	0	0	0	0	0	N
Delcer	Black	River	4-Jul-22	13.78704	-61.0605	0	0	P	0	P	0	0	0	N
Desruisseaux	Black	Stream	4-Jul-22	13.79738	-60.9449	0	0	P	0	0	0	0	0	N
Desruisseaux2	Black	River	4-Jul-22	13.80179	-60.9532	0	0	P	0	0	0	0	0	N
Derachè Stream	Black	Stream	30-Jun-22	13.84094	-61.0168	0	0	0	0	0	0	0	0	N
Fond Assau*	Black	Stream	28-Jun-22	13.99623	-60.9315	0	P	P	0	0	0	0	0	N
Garrand-Babonneau*	Yellow	River	28-Jun-22	14.00454	-60.9202	*kuhniana* (3)	P	P	0	0	0	0	0	Y
Jacmel Ditch	Black	Ditch	28-Jun-22	13.95022	-61.016	0	0	P	0	0	0	0	0	N

Jacmel Gutter	Yellow	Drainage Gutter	28-Jun-22	13.94996	-61.0159	*kuhniana* (8)	0	P	0	0	0	0	0	Y
Jacmel Stream	Black	Stream	28-Jun-22	13.94599	-61.0132	0	4	P	0	0	0	0	0	N
La Ressource Gutter*	Black	Gutter	30-Jun-22	13.94199	-60.914	*kuhniana* (shells only)	15	P	0	0	0	0	0	Y
Laborie*	Yellow	Water catchment	4-Jul-22	13.74631	-60.9854	*kuhniana* (1)	5	P	120	0	0	0	0	Y
Latille Falls Micoud	Yellow	Waterfall	30-Jun-22	13.8325	-60.9188	*kuhniana* (1)	3	P	0	0	0	0	0	Y
Leriche Stream	Black	River	4-Jul-22	13.78106	-61.0543	0	0	P	0	0	0	0	0	N
Micoud South*	Black	Stream	30-Jun-22	13.82527	-60.9049	0	0	0	0	0	0	0	0	N
Micoud Stream*	Black	Stream	30-Jun-22	13.82597	-60.9062	0	0	P	0	4	0	0	0	N
Migny Banana Ravine	Black	Ravine	30-Jun-22	13.83715	-61.0185	0	0	P	0	0	0	0	0	N
Millet Bridge*	Black	Stream	28-Jun-22	13.91614	-60.9931	0	0	P	0	0	0	0	0	N
Millet Duarandeau*	Black	Stream	28-Jun-22	13.92616	-60.9925	0	0	P	0	0	0	0	0	Y
Moison Stream	Black	Stream	4-Jul-22	13.78299	-61.056	0	0	P	0	0	0	0	0	N

					2									
Monier*	Black	Stream	28-Jun-22	13.99378	-60.9353	0	5	P	0	0	0	0	0	N
Morne D'Or	Black	Stream	28-Jun-22	13.95042	-61.0052	0	0	P	0	0	0	0	0	N
Piaye Bridge	Black	River	4-Jul-22	13.75846	-61.0205	0	0	P	0	0	0	0	0	N
Ravine Poisson	Black	River	28-Jun-22	13.92804	-60.9715	0	0	P	0	0	0	0	0	N
Sarot Pond 1	Black	Pond	28-Jun-22	13.94394	-60.9902	0	0	P	0	0	0	0	0	Y
Sarot Pond 2	Black	Pond	28-Jun-22	13.94394	-60.9902	0	0	P	0	0	0	0	0	N
Sarot Pond 3	Yellow	Pond	28-Jun-22	13.94424	-60.9898	kuhniana (30)	13	P	0	0	0	0	0	Y
Sarot River	Black	River	28-Jun-22	13.94504	-60.9912	0	0	P	0	0	0	0	0	Y
St. Phillips Stream*	Black	Stream	30-Jun-22	13.84768	-61.028	0	11	P	0	0	0	0	1	N
Ti Boug	Black	Stream	30-Jun-22	13.83645	-61.0224	0	P	0	0	0	0	0	0	N
Union Ministry Health	Yellow	Pond	30-Jun-22	14.07425	-60.9311	kuhniana (1)	2	P	0	0	16	0	0	Y

Vannard	Black	River	28-Jun-22	13.94481	-60.9909	0	P	P	0	0	0	0	0	N
Vieux Fort, Beanfield Rd*	Black	Pond	4-Jul-22	13.77203	-60.9162	0	0	P	0	0	0	0	0	Y
Zenon Stream*	Black	River	28-Jun-22	13.85632	-61.0362	0	0	0	0	0	0	0	0	N
*Sites that were nearby sites or the same sites reported in Pointier et al., 1993														
